# Advancements in the treatment of cerebral ischemia-reperfusion injury: Acupuncture combined with mesenchymal stem cells transplantation

**DOI:** 10.1097/MD.0000000000041075

**Published:** 2025-01-10

**Authors:** Huan Li, Jiaxin Zhang, Kewen Ma, Jie Ji, Chengfei An, Hailun Jiang, Hui Qu, Ruohan Tang, Xuesong Ren, Yuzheng Du, Qi Zhao

**Affiliations:** a First Teaching Hospital of Tianjin University of Traditional Chinese Medicine, Tianjin, China; b National Clinical Research Center for Chinese Medicine Acupuncture and Moxibustion, Tianjin, China; c Tianjin University of Traditional Chinese Medicine, Tianjin, China; d The First Affiliated Hospital of Henan University of Traditional Chinese Medicine, Zhengzhou, China.

**Keywords:** acupuncture, cerebral ischemia-reperfusion injury, electroacupuncture, mesenchymal stem cell, neuromodulation, stroke

## Abstract

Cerebral ischemia-reperfusion injury (CIRI) constitutes a significant etiology of exacerbated cerebral tissue damage subsequent to intravenous thrombolysis and endovascular mechanical thrombectomy in patients diagnosed with acute ischemic stroke. The treatment of CIRI has been extensively investigated through a multitude of clinical studies. Acupuncture has been demonstrated to be effective in treating CIRI. Recent 5 years studies have identified potential mechanisms of acupuncture, including regulation of autophagy, promotion of angiogenesis, inhibition of inflammation and apoptosis, modulation of cell activation, neuroplasticity regulation, and promotion of nerve regeneration. The transplantation of mesenchymal stem cells (MSCs) can effectively suppress apoptosis, modulate immune responses, and enhance the proliferation and migration of endogenous neural stem cells (NSCs), thereby compensating for the NSCs deficiency following cerebral ischemia/reperfusion injury. The combination of acupuncture and MSCs transplantation demonstrates superiority over individual treatments, significantly enhancing the survival rate of MSCs. Moreover, it facilitates the secretion of various cytokines to promote their homing and differentiation into functional neurons, thereby providing a novel approach for clinical treatment of CIRI.

## 1. Introduction

The incidence, prevalence, disability, recurrence, and mortality rates of stroke are all significantly elevated, representing a formidable threat to public health and imposing substantial economic burdens on both families and society. Among them, ischemic stroke constitutes 75% to 80% of the overall stroke patient population.^[1]^ Ischemic stroke is the result of blood circulation disorders that lead to vascular occlusion or severe stenosis, resulting in reduced cerebral blood perfusion, local cerebral tissue ischemia, hypoxia and ultimately neuronal death, which triggers corresponding neurological deficits.^[2]^ Early administration of thrombolytic agents and endovascular mechanical thrombectomy are established strategies for restoring cerebral blood flow.^[3,4]^ However, cerebral ischemic regions undergoing ischemia and reperfusion will initiate a cascade of intricate changes, including oxidative stress, uncontrolled secretion of inflammatory mediators, leukocyte infiltration, disruption of the blood-brain barrier, microcirculatory disorders, and mitochondrial dysfunction. These changes ultimately lead to neuronal death and neurological deficits while exacerbating damage to brain tissue.^[5–11]^ Hence, the advancement of safer and more efficacious therapies is crucial in enhancing clinical effectiveness.

Acupuncture, an integral component of traditional Chinese medicine, involves the insertion of fine metal needles into specific acupoints to prevent and treat diseases through targeted techniques or enhanced stimulation that restores bodily equilibrium.^[12,13]^ Studies have shown that acupuncture activates axonal regeneration and sprouting, stimulates neurogenesis, enhances neural plasticity, regulates neurotransmitter activity, attenuates oxidative stress, inhibits apoptosis and autophagy, suppresses inflammatory responses, improves cerebral blood flow, and reduces cerebral edema to alter neural networks and functions in damaged brain regions, thus helping post-stroke patients to regain a level of daily life function.^[14,15]^ However, the application of acupuncture for nerve regeneration still faces obstacles due to insufficient numbers of endogenous neural stem cells (NSCs) and neural precursor cells, which are necessary for sustaining neurological function recovery after ischemic stroke. Therefore, further research is required to advance progress in this field.

The “mesenchymal stem cells” (MSCs) are derived from embryonic mesoderm, and they are attractive cells because of their proliferative capacity, multidirectional differentiation, and immunomodulatory properties.^[16,17]^ MSCs possess migratory and homing capabilities, allowing them to migrate towards damaged tissues during development and after systemic infusion, differentiate into functional cells with reparative therapeutic effects, and ultimately regenerate the affected tissues.^[18,19]^ In addition to expressing various neurotrophic factors, MSCs can facilitate functional rehabilitation by promoting angiogenesis, reducing neuronal apoptosis, reconstructing synapses and dendrites, as well as facilitating axonal regeneration and differentiation of NSCs.^[20]^ It has been reported that MSCs exert protective effects through paracrine signaling, whereby the resulting extracellular vesicles contain a diverse array of soluble factors including growth factors, chemokines, cytokines and even microRNAs. These factors inhibit the up-regulation of pro-inflammatory mediators while promoting anti-inflammatory and regenerative effects.^[21]^ Both clinical and basic studies have shown that MSCs transplantation is effective in CIRI, which can improve cerebral ischemia, reduce the area of cerebral infarction and the number of apoptotic cells, and better promote brain recovery.^[22,23]^ Despite the numerous advantages of MSCs transplantation therapy, it still faces challenges such as limited differentiation potential, short half-life duration, immunosuppressive effects, restricted targeting abilities, inefficient payload transportation and rapid post-application clearance.^[24,25]^ Recently, a plethora of domestic and international research investigations have unequivocally demonstrated the remarkable efficacy of acupuncture in conjunction with MSC transplantation for patients suffering from CIRI. Therefore, this study presents a comprehensive analysis of recent publications to investigate the clinical curative effect and underlying mechanisms of acupuncture as a standalone intervention or in combination with MSC transplantation for the treatment of CIRI. The objective is to critically assess the potential evidence and offer novel insights into the clinical application of acupuncture in managing CIRI.

## 2. Effectiveness and mechanisms of acupuncture in CIRI

### 2.1. The clinical effectiveness of acupuncture in neurorehabilitation for patients with CIRI

The effectiveness of acupuncture in improving neurological and motor function in patients with CIRI has been demonstrated through multiple randomized controlled trials. The study conducted by Zhan et al^[26]^ revealed that acupuncture holds promise in augmenting neurological function and enhancing performance in activities of daily living among patients who have received thrombolysis for acute ischemic stroke. Meanwhile, acupuncture treated 71 patients after cerebral infarction with recombinant tissue-type fibrinogen activator intravenous thrombolysis, and the results showed that acupuncture could reduce symptomatic intracranial hemorrhage and the incidence of adverse reactions.^[27]^ Meta-analysis showed that combining acupuncture with thrombolysis for acute cerebral infarction had a higher efficiency; and the neurological function and the ability of daily autonomous life were both significantly improved.^[28]^ The meta-analysis mentioned above still has some limitations, including the absence of high-quality multicenter large-sample trials, the lack of a standardized approach to acupuncture, and potential biases in clinical trial design. Therefore, while acupuncture has shown effective improvement in various complications after CIRI, further research with rigorous methodologies is needed. Therefore, in the future, more multi-center, high-quality clinical trials and clear standardization of acupuncture practices may be helpful to evaluate and validate its effectiveness and clinical results.

### 2.2. Elucidating the mechanisms that underlie acupuncture in CIRI

The CIRI can be categorized into 3 distinct stages: acute, subacute, and chronic. During the acute phase following an ischemic stroke, there is a decrease in cerebral perfusion and an immediate cessation of oxygen and glucose supply. This leads to subsequent recirculation of cerebral ischemia, which triggers increased expression of inflammatory vesicles by immune cells and neuroglia, ultimately resulting in disruption of the blood-brain barrier.^[29]^ In the subacute phase, reactive oxygen species induce apoptosis and upregulate adhesion molecule expression, activate microglia, promote leukocyte release of pro-inflammatory mediators, and cause damage to the blood-brain barrier and endothelial cells.^[30]^ During the chronic phase, neural stem cells demonstrate migratory behavior towards the peri-infarct region and undergo neuronal differentiation. The process of neural stem cell proliferation is characterized by the secretion of insulin-like growth factor, glial cell-derived neurotrophic factor (GDNF), and brain-derived neurotrophic factor (BDNF). Nevertheless, in an inflammatory environment lacking trophic factors, it is possible that the viability of a substantial number of immature neurons could be jeopardized.^[31]^ Cerebral ischemia and reperfusion can lead to secondary brain injury such as cerebral edema, parenchymal hemorrhage, and vascular occlusion due to deleterious free radical production, apoptosis, endothelial cell damage, and destruction of neurovascular units.^[32,33]^ Studies in the last 5 years have shown that acupuncture can alleviate CIRI by regulating autophagy, promoting angiogenesis, inhibiting inflammation, inhibiting apoptosis, regulating cell activation, modulating neuroplasticity, and promoting nerve regeneration (Table 1). The potential mechanisms by which acupuncture, as a complementary and alternative therapy, stimulates the peripheral posterior and then modulates the cerebral centers to promote neural recovery and functional restoration after CIRI are as follows.

**Table 1:** Mechanisms underlying the effects of acupuncture on cerebral ischemia-reperfusion injury repair.

Study	Acupuncture intervention	Control intervention	Effect indicators	Mechanism index
Acupuncture regulates autophagy				
[34]	Acupuncture (GV14, GV26, GV20; 30 min, once every 12 h for a total of 7 times)	Not received EA treatment	Garcia’s score↑, the percentage of cerebralis chemicarea ↓	III PI3K↑, Beclin-1↑, LC3B-II/I↑, Lamp2↑, P62↓, type III PI3K/Beclin-1 pathway↑
[35]	Acupuncture (liver, upper energy, lower energy, kidney; scrape the needle 5 times every 10 min, 30 min each time)	Not received EA treatment	The Longa’s score↓, areas of typical ethmoidal reticular cerebral infarction↓, autophagosomes↓	LC3B↓, Beclin1↓, ATF6↓, XBP1↓, ATF6 pathway↓
[36]	EA (GV24, GV20; 1–20 Hz, 2 mA, 6 V, 30 minutes, 8 d)	Not received EA treatment	The Longa Score Scale↓, the infarct volume↓, cell apoptosis↓	Beclin-1↑, PI3K↑, mTOR↑, p53↓, PI3K/Akt signaling pathway↑
[37]	EA (GV20, GB7; 2–20 Hz, 30 min every 12 , 3 d)	I: electroacupuncture with a nonacupoint II: not received EA treatment	The infarct volumes↓, neurological score↓	GRP78↓, ATF6↓, ATF-4↓, CHOP↓, p-PERK/PERK↓, p-IRE1/IRE1↓, and p-eIF2α/eIF2α ↓, ATF6↓, p-PERK↓, p-IRE1↓, LC3↓, p62↑, the ratio of LC3II/I and Beclin-1 protein↓, Bax↓, cleaved caspase-12↓, cleaved caspase-9↓, cleaved caspase-3↓, cleaved PARP↓, Bcl-2↑, ATF6, PERK, and IRE1 pathways↓
[38]	Acupuncture (GV14, GV20, GV26; simulated manually once every 15 min, 30 min)	Not received EA treatment	Garcia’s core↑, the percentage of cerebral infarct area↓, the apoptosis rate↓, the damage of neurons↓	miR-34c-5p↑, Beclin1 p62↑, LC3B-II↑, LC3B-I↑
[39]	EA (DU24, GV20; 4 Hz/20 Hz, 0.5 mA, 2 V, 30 min, 7 d)	I: not received EA treatment II: inhibitor group: EA + luzindole	The cerebral infarct volume↓, the route and escape latency↓, the number of crossing the platform position↑, ultrastructural changes in the mitochondria↓	Melatonin↑, AANAT↑, the LC3-II/I ratio↑, PINK1↑, Parkin↑, ROS↓, NLRP3 ↓, IL-1β↓, IL-18↓, Iba-1-positive cells↓
[40]	Acupuncture (liver, upper energy, lower energy, kidney; 0.5 h, 12 h and 24 h after the modeling; 30 min)	Not received EA treatment	Zea Longa score↓, blood flow volume and blood flow speed↑, the number of Nissl bodies↑	Beclin-1 protein↓, LC3-II/ LC3-I↓, p62↑
[41]	EA (GV20, ST36; 20 Hz, 30 min)	Not received EA treatment	The infarct size↓, Garcia JH scoring↑, the accumulation of damaged mitochondria↓	MMP↑, ATP↑, Mfn1↑, OPA1↑, COX IV↓, VDAC↓ TOMM20↓, NOX↓, ROS↓, MDA↓, SOD↑, iNOS↓, 3-NT↓, mTOR↓, Rab7↓, p62↓, LAMP-2↑, LC3↑, Drp1↑, Parkin↑, Mfn2↑, Nissl + neurons↑
[42]	EA (GV20, GV26; 3.85 Hz for 1.28 s and 6.25 Hz for 2.08 s, 0.8–1.0 mA, 30 min)	No treatment	The Longa test↓, the infarct volume↓	LC3-II↑, Beclin1↑, hMOF↓, H4K16ac↓, Sirt1↑
Acupuncture promotes angiogenesis				
[43]	EA (GV26, PC6, SP6; 2/15 Hz, 1 mA, 20 min)	Not received EA treatment	Neurological deficit score↓, the volume of cerebral infarction↓, ultrastructure of vascular ECs↑	VEGFA↑, Notch1↑, the downstream target protein Hes1↑, the VEGF/notch pathway↑
[44]	EA (GV26; 15 Hz, 1 mA, 20 min)	Not received EA treatment	The cerebral infarct volume↓, Bederson score↓, the viability of BMECs↑	CD34↑, EPO↑, VEGF↑, p-Src↑, EPO-mediated Src and VEGF signaling pathways↑
[45]	Scalp acupuncture group (GV20, the 5 equal points around the center of the circle; 3–15 Hz, 2–4 mA, 30 min)	Not received EA treatment	mNSS↓, the infarcted areas↓, rCBF↑, the number of nerve cells↑	Wnt3a↑, β-Catenin↑, TCF4↑, Cyclin D1↑, p-GSK3β↓, VEGF↑, FLK1↑, bFGF↑, Ang2↑, Wnt/β-Catenin signal pathway↑
[46]	EA (GV26, GV20; 0.8–1.3 mA, a density wave was used, 3.85 Hz, 1.28 s; a dense wave frequency, 6.25 Hz, 2.08 s; 30 min, once a day, 3 d)	Not received EA treatment	Longa’s neurologic deficit scoring↓, infarct volume↓, neuronal cell necrosis↓, cell shrinkage↓, chromatin condensation and fragmentation↓	miR-210↑, CD34↑, HIF-1α↑, VEGF↑, Notch1↑, the HIF1α/VEGF/Notch1 signal pathway↑
Acupuncture inhibits inflammation				
[47]	EA (TE5, ST36; 20 HZ, 1 mA, 30 min, 7 d)	Not received EA treatment	mNSS↓, the infarct volume↓, TUNEL-positive neurons↓, the number of viable neurons↑	miR-223↑, NLRP3↓, caspase-1↓, IL-1b↓, IL-18↓, miR-223/NLRP3 pathway↓
[48]	EA (GV26, PC6, SP6, BL40; 1–20 Hz, 6 V, 30 min, 28 times)	Not received EA treatment	Zea Longa score↓	TLR2↓NF-kB↓, IRAK↑, TLR2/NF-κB signaling↓
[49]	EA (GV20, 2/15 Hz, 1 mA, 20 min)	Not received EA treatment	Longa neurologic score↓, the cerebral infarction area↓	TNF-α↓, IL-1β↓, IL-10↑
[50]	EA (GV20, 2/15 Hz, 1 mA, 20 min)	Not received EA treatment	The Longa neurological scale↓, the infarcted area↓, the percentage of Tregs cells↑, the percentage of γδ T cells↓, the percentage of CD4^+^ T cells↓, the percentage of TCRγδ^+^ T cells↓	TNF-α↓, IL-1β↓, Cxcl1↓, Cxcl2↓, IL-10↑, DAO↓, D-LAC↓, ZO-1↑, Occludin↑, Claudin-1↑
[51]	EA (GV20, DU24; 0.05 Hz, 6 V, 30 min, 7 d)	IC: not received EA treatment NA: EA on torso	Neurological deficit scores↓, the infarct volume↓, the histopathological in the hippocampus of I/R rats↑, learning and memory function↑	Leukocyte common antigen/ CD45↓, NF-κB p65↓, IκBα↑, TNF-α↓, IL6↓, IL-1b↓
[52]	Acupuncture (GV14, GV20, GV26, ST36, GB20; 20 min, once a day, 7 d)	Not received EA treatment	Longa’s score↓	NeuN↑, Iba-1↓, iNOS↓, Arg1↑, BDNF↑, GDNF↑, TNF-α↓, IL-6↓, IL-10↑, BDNF↑, GDNF↑, TLR4↓, MyD88↓, NF-κB ↓, TLR4/MyD88/NF-κB signaling pathway↓
[53]	EA (LU5, LI4, ST36, SP6; 5/10 Hz, 2 mA, 20 min)	Not received EA treatment	Neurological deficit scores↓, the volume of cerebral infarction↓	NLRP3↓, pro-Casp-1↓, Casp-1 p20↓, IL-1β↓, cleaved IL-1β↓, GSDMD↓
[54]	EA (GV20, GV24; 4/20 Hz, 0.5 mA, 20 min)	I: not received EA treatment II: EA + Melatonin receptor inhibitor Luzindole	The neural function score↓, the percentage of cerebral infarction volume↓, apoptosis rate of nerve cells in cerebral cortex area of infarction side↓	The melatonin↑, the activation level of microglia cells↓, NLRP3↓, Caspase-1↓, IL-1β↓,
Acupuncture inhibits cell apoptosis and activation				
[55]	EA (GV20, GV24; 4/20 Hz, 2 V, 0.5 mA, 20 min, once a day, 7 d)	I: not received EA treatment II: non acupoints below the costal margins on both sides	Longa’s score↓, the escape latency↓, the times of platform quadrant crossing↑	The secretion of melatonin 24:00↑, AANAT↑, NeuN protein↑, GFAP↓
[56]	EA (LI4, LU5, ST36, SP6, 2–10 Hz, 3–5 V, 20 min)	I: not received EA treatment II: caspase-3 inhibitor groups	Infarct volume↓, the apoptosis dex↓, the neurological deficit scores↓	Caspase-3↓
[57]	Acupuncture (liver regions, kidney regions, upper-energizer, lower-energizer, 20 min, once every 12 h)	Not received EA treatment	Neural function score↓	Nestin/TUNEL↓, CD34/TUNEL↓, GFAP/TUNEL↓, Bad↓, Bcl-xL↑
[58]	EA (LI11, ST36; 2/15 Hz, 2–4 mA, 20 min, 7 d)	Not received EA treatment	The Longa score↓, the balance beam score↓, the percentage of cerebral infarction volume↓	PGC-1α↑, FNDC5↑, BDNF↑, PGC-1α/Irisin (FNDC5)/BDNF pathway↑
[59]	EA (GV20, EX-HN3, ST36; 4/20 Hz, 10 min, 7 d)	Not received EA treatment	Longa’s score↓, gait dynamics↑, the morphological and structural changes in the brain tissues↑	GFAP↑, PI3K↑, the PI3K/AKT signaling pathway↑
Acupuncture promotes nerve regeneration and regulates Synaptic plasticity				
[60]	EA (GV20, ST36; 2 Hz, 1 mA, 30 min)	Not received EA treatment	The infarct size↓, the Garcia JH score↑, the ultrastructural injury of the hippocampus↓	GAP43↑, BDNF↑, OMgp↓, NogoA↓, NgR↓, MAG protein↑, MAG mRNA↓, ROCK2↓, MYPT1↓, MLC1↓, RhoA↓, ROCK mRNA↓, MLC mRNA↓, MYPT1 mRNA↓, RhoA/ROCK pathway↓
[61]	EA (EX-HN3, GV20; 2 Hz, 1 mA, 100 μs, 10 min, 14 d)	Not received EA treatment	Learning and memory↑	NMDAR1↑, AMPAR↑, GABAAR↑, CaMKII↑, NeuN↑, PSD-95↑, BNDF↑, TRKB↑
[62]	EA (TE5, ST36; 20 Hz, 1 mA, 30 min, 7 d)	I: not received EA treatment II: nonacupoints	mNSS↓, the volume of cerebral infarction↓	miR-223↑, PTEN↓, Nestin↑, Notch1↑, NOTCH1/mir-223/PTEN pathways↑

3-NT = the level of 3-nitrotyrosine, AANAT = arylalkylamine N-acetyltransferase, AKT = phosphoprotein kinase B, AMPAR = α-amino-3-hydroxy-5-methyl-4-isoxazole propionic, Arg1 = Arginase 1, ATF-4 = transcription factor 4, ATF6 = activating transcription factor 6, Bax = Bcl-2 Associated X Protein, Bcl-2 = B-cell lymphoma-2, BDNF = brain-derived neurotrophic factor, BL40 = Weizhong, BMECs = brain microvascular endothelial cells, CaMKII = Ca^2+^/calmodulin-dependent protein kinase II, CD34 = cluster of differentiation 34, CD4+ T cells = CD4^+^ positive cells, CHOP = C/EBP-homologous protein, COX IV = cytochrome c oxidase IV, DAO = diamine oxidase, D-LAC = D-lactate, DU24 = Shenting, eIF2α = α unit of eukaryotic initiation factor 2, EPO = erythropoietin, EX-HN3 = Yintang, FNDC5 = fibronectin type III domain-containing protein5, GABAAR = acid receptor and γ-aminobutyric acid type A receptor, GB20 = Fengchi, GB7 = Qubin, GDNF = glial cell line-derived neurotrophic factor, GRP78 = glucose-regulated protein 78, GV14 = Dazhui, GV20 = Baihui, GV24 = Chengjiang, GV26 = Shuigou, Iba-1 = ionic calcium-binding protein 1, III PI3K = type III photoshatidylinositol3-hydroxykinase, IL-10 = interleukin-10, interleukin-18, IL-1β = interleukin-1β, IL-6 = interleukin-6, iNOS = inducible nitric oxide synthase, IRAK = interleukin-1 receptor-associated kinase, IRE1 = inositol-requiring protein 1, Lamp2 = lysosome associated membrane protein2, LAMP-2 = lysosome-associated membrane protein type 2, LC3B = microtubule-associated protein1 light chain 3B, LI11 = Quchi, LI4 = Hegu, LU5 = Chize, MAG = myelinassociated glycoprotein, MDA = malondialdehyde, Mfn = mitofusin, OPA1 = optic atrophy 1 gene protein, miR-210 = micro RNA-210, miR-223 = microRNA-223, MMP = mitochondrial membrane potential, mNSS = modified neurological severity score, mTOR = mammalian target of rapamycin, MyD88 = medullary differentiation factor 88, MYPT1 = myosin phosphatase target subunit-1, NeuN = neuron-specific nuclear protein, NF-κB = nuclear factor kappa B, NgR = Nogo receptor, NLRP3 = nucleotide-binding domain-like receptor family, NMDAR = N-methyl-D-aspartate receptor, NogoA = neurite outgrowth inhibitor A, NOX = oxidase, OMgp = oligodendrocyte-myelin glycoprotein, PARP = cleaved poly ADP-ribose polymerase, PC6 = Neiguan, PERK = PKR-like ER kinase, PGC-1α = peroxisome proliferator-activated receptor gamma coactivators-1 alpha, PI3K = phosphoinositide 3-kinase, PSD-95 = postsynaptic density protein 95, p-Src = phospho-Src, rCBF = regional cerebral blood flow, ROS = reactive oxygen species, SOD = superoxide dismutase, SP6 = Sanyinjiao, ST36 = Zusanli, TE5 = Waiguan, the percentage of γδ T cells, TLR2 = toll like receptor 2, TLR4 = toll like receptor 4, TNF-α = tumor necrosis factor-α, TOMM20 = translocase of outer mitochondrial membrane 20 homolog, Tregs = regulatory T cells, TRKB = tyrosine kinase B, VDAC = voltage dependent anion channel, VEGF = vascular endothelial growth factor, XBP1 = X-box binding protein 1.

#### 2.2.1. Acupuncture regulates autophagy

Autophagy eliminates damaged organelles and misfolded proteins, thereby safeguarding normal cellular functions and promoting cell survival. Although the role of autophagy in ischemic stroke remains a topic of debate, it is widely acknowledged that moderate levels of autophagy can confer a protective effect.^[40,63–65]^ Acupuncture has been demonstrated to regulate autoregulation dependent on endoplasmic reticulum stress by activating relevant signaling pathways, including phosphatidylinositol 3-kinase (PI3K)/protein kinase B (Akt), PKR-like ER kinase (PERK), activating transcription factor 6 (ATF6) and inositol-requiring protein 1 (IRE1).^[34,35,38,42]^ Regulating autophagy triggered by endoplasmic reticulum stress and mitochondrial dysfunction, with the aim of mitigating neurological damage following reperfusion injury, involves activation of the Akt, PERK, IRE1 endoplasmic reticulum membrane protein, and ATF6 signaling pathways.

The PI3K/Akt pathway inhibits the cellularization of nerve growth factor in ischemic stroke, thereby reducing the extent of tissue damage and improving neurological prognosis. Meanwhile, autophagy exerts a neuroprotective effect by suppressing mammalian target of rapamycin (mTOR) activity.^[66,67]^ Wang et al^[36]^ demonstrated that electroacupuncture (EA) targeting *Chengjiang* and *Baihui (GV20*) can elicit neuroprotective effects through activation of the PI3K/Akt signaling pathway, upregulation of PI3K, mTOR, Beclin-1 protein and mRNA expression levels, downregulation of p53 expression level, as well as induction and enhancement of autophagy.

The unfolded protein response (UPR) is a signaling mechanism that is triggered by endoplasmic reticulum stress. It encompasses 3 downstream-regulated signaling pathways, namely PERK, ATF6, and IRE1. Upon activation, these pathways orchestrate remodeling of the Endoplasmic Reticulum quality control to mitigate cellular stress and restore endoplasmic reticulum function.^[68]^ It has been demonstrated that acupuncture targeting *GV20* and Qubin effectively attenuated endoplasmic reticulum stress in cerebral ischemia-reperfusion injury (CIRI) by modulating the PERK, IRE1, and ATF6 signaling pathways. This was evidenced by significant downregulation of ATF6, p-PERK, and pIRE1 expression levels, as well as inhibition of ER stress-induced autophagy, ultimately leading to alleviation of CIRI.^[37]^

The administration of melatonin triggers the activation of host defense mechanisms, modulates multiple signaling pathways to safeguard against NOD-, LRR- and pyrin domain-containing protein 3 (NLRP3) inflammatory vesicle-induced injury, diminishes the formation of reactive oxygen species (ROS), promotes detoxification processes, and shields against mitochondrial damage.^[69]^ The neuroprotective effects of EA at *Shenting (DU24*) and *GV20* are attributed to the regulation of endogenous melatonin, upregulation of mitochondrial autophagy-related proteins, activation of mitochondrial autophagy, preservation of mitochondrial integrity, and reduction in ischemia/reperfusion-induced mitochondrial damage and ROS generation.^[39]^ PTEN-induced kinase 1 (PINK1) and Parkin RBR E3 ubiquitin-protein ligase (PARKIN) play crucial roles in the targeted removal of dysfunctional or superfluous mitochondria, thereby finely sustaining energy metabolism and regulating the mitochondrial network. PINK1 governs the translocation of PARKIN to damaged mitochondria and initiates its clearance via mitophagy. Modulation of the Pink1/Parkin signaling pathway confers cellular protection against mitochondrial stress-induced dysfunction.^[70]^ The application of EA at *GV20* and *Zusanli (ST36*) alleviates nitro/oxidative stress-induced mitochondrial dysfunction, enhances the elimination of damaged mitochondria through Pink1/Parkin-mediated mitophagy, and mitigates neurological damage during cerebral ischemia/reperfusion.^[41]^

In summary, the current acupuncture treatment for CIRI involves modulating multiple pathways to achieve moderate regulation of autophagy and exert a neuroprotective effect. This aligns with the comprehensive regulatory characteristics of acupuncture, which encompass engagement with multiple targets, repeated sessions, involvement of multiple pathways, and regulation at various levels. The modulation of acupuncture on autophagy may exert distinct effects at various pathological phases of CIRI, and further investigation is necessary to elucidate the evidence-based rationale for enhancing its beneficial impacts.

#### 2.2.2. Acupuncture promotes angiogenesis

Angiogenesis plays a pivotal role in the remodeling of the neurovascular unit following an ischemic stroke, not only facilitating improved oxygenation and nutrition supply to the cerebral ischemic penumbra and restoration of blood flow to the affected region but also promoting timely recovery of damaged brain tissues and neurological functions.^[71]^ Acupuncture has the potential to augment angiogenic factor expression, safeguard vascular endothelial cells, and stimulate angiogenesis via signaling pathways involving Wnt/β-catenin, vascular endothelial growth factor (VEGF), Src, Notch, and HIF-1α.

The Src family of tyrosine kinases is intricately associated with an intracellular signaling pathway that governs the modulation of cell surface receptors in diverse ways. As a result, this mechanism facilitates cellular growth, proliferation, adhesion, and motility functions. Additionally, Src plays a critical role in angiogenesis through various signaling pathways.^[72]^ Our findings demonstrate that EA at *Shuigou (GV26*) enhances angiogenesis by activating the erythropoietin (EPO)-mediated Src and VEGF signaling pathways, resulting in increased expression of EPO and VEGF factors that mitigate CIRI.^[73]^ The Notch signaling pathway plays a crucial role in regulating angiogenesis by controlling various processes, such as endothelial cell proliferation, differentiation, migration, and adhesion.^[44]^ The EA stimulation of *Neiguan (PC6*), *GV26*, and *Sanyinjiao (SP6*) can induce the activation of the VEGF/Notch signaling pathway, resulting in an upregulation of Hes1 expression, a downstream target protein.^[74]^

The Wnt/β-catenin signaling pathway is crucially involved in the process of angiogenesis, and the utilization of inhibitors targeting this pathway has demonstrated remarkably potent anti-angiogenic effects. Furthermore, sustained activation of the Wnt/β-catenin signaling cascade during angiogenesis results in an upregulation of pro-angiogenic factors.^[43]^ The application of acupuncture can activate the Wnt/β-catenin signaling pathway, thereby enhancing the expression of angiogenic factors such as VEGF, fetal liver kinase 1, basic fibroblast growth factor (bFGF), and Angiopoietin-2 in brain tissue following injury. This restoration of blood flow and perfusion in the ischemic area contributes to a reduction in the size of the infarcted region.^[75]^

HIF-1α plays a pivotal role in cerebral ischemia, and its activation during ischemic events exerts a protective effect on the affected tissues by participating in metabolic, proliferative, and angiogenic processes.^[45]^ The EA treatment induced activation of the HIF-1α/VEGF/Notch1 signaling pathway via exosomal micro RNA-210, resulting in increased expression of cluster of differentiation 34, HIF-1α, VEGF, and Notch1 at both protein and mRNA levels. Ultimately, this facilitated angiogenesis in the context of ischemic stroke.^[76]^

The above studies pointed out that acupuncture can regulate angiogenic factors and pathway proteins, reduce the damage of vascular endothelial cells, and then promote angiogenesis, which involves complex and diverse mechanisms, and the effect of EA may be the superimposed effect of a variety of factors, and it is not possible to clarify the role of a specific single factor. Moreover, most of the studies focused on the ischemic side of the infarcted area, and the changes in the microvascular network of acupuncture in promoting angiogenesis on the healthy side, as well as the mechanisms at the molecular level still need to be further elucidated.

#### 2.2.3. Acupuncture suppresses inflammation

Reperfusion subsequent to ischemia is increasingly acknowledged as a trigger for local inflammation, which in turn leads to augmented brain damage and neuronal cell demise through intricate molecular pathways. Ultimately, this culminates in CIRI, primarily characterized by apoptosis, necrosis, and autophagy.^[46]^ Therefore, reducing neuroinflammation is important for alleviating neurological injury. The application of acupuncture has the potential to mitigate neuroinflammation following CIRI through the downregulation of inflammatory and neurotrophic factors, as well as the suppression of pro-inflammatory mediators.

The expression of microRNA-223 (miR-223), a multifunctional miRNA, is tightly regulated by multiple transcription factors and exhibits a significant upregulation in the context of cellular or tissue inflammation.^[77]^ Upon activation of caspase-1, a cascade of reactions is initiated resulting in the release of pro-inflammatory cytokines such as interleukin-1β (IL-1β) and interleukin-18 (IL-18). This can give rise to a spectrum of conditions ranging from infections to inflammation to autoimmune disorders.^[78]^ The EA treatment demonstrates the potential to mitigate neuroinflammation and exert neuroprotective effects through the inhibition of the miR-223/NLRP3 pathway, upregulation of miR-223 expression, and downregulation ofcaspase-1, NLRP3, IL-18, as well as IL-1β levels.^[79]^

The nuclear factor kappaB (NF-κB) governs immune and inflammatory processes, playing a pivotal role in orchestrating the transcriptional activation of genes associated with immune and inflammatory responses.^[47]^ EA at *GV20* and *DU24* exerts a suppressive effect on the expression of NF-κB p65, leukocyte common antigen/CD45, interleukin-6 (IL-6), IL-1β, and tumor necrosis factor-alpha (TNF-α) in hippocampal CA1. This inhibits NF-kB-mediated inflammatory damage and ameliorates post-stroke memory and learning deficits.^[80]^ Toll-like receptor 2 (TLR2) plays a pivotal role in the cascade of innate immunity within the brain, and its involvement in ischemic stroke is critical. The observed reduction in central nervous system damage in TLR2-deficient mice with focal ischemia suggests that TLR2 exerts detrimental effects by promoting inflammation during the occurrence of an ischemic stroke.^[51,78]^ EA stimulation of *GV26*, *PC6*, *SP6*, and *Weizhong (BL40*) acupoints exerts inhibitory effects on the TLR2/NF-κB signaling pathway, leading to downregulation of TLR2 and NF-κB expression. Additionally, it delays the peak expression of IL-1β receptor-associated kinase (IRAK), thereby improving neurological deficits following CIRI.^[81]^

Following acute central nervous systems (CNS) injury, alterations in the composition of intestinal microbiota disrupt the bidirectional gut-brain axis balance, leading to an inflammatory response in both peripheral and CNS, ultimately resulting in a poor prognosis.^[48]^ The balance between regulatory T cells and γδ T cells in both the brain and small intestine can be modulated by EA, thereby exerting inhibitory effects on ischemic inflammation and mitigating CIRI.^[49,82]^ The Myeloid differentiation primary response 88 (MyD88) serves as a pivotal hub in the inflammatory pathway, facilitating signal transmission from members of the Toll-like receptor family to kinases in the IRAK family via interactions between proteins of the same type. Subsequent activation of IRAK family kinases elicits diverse functional outcomes, including NF-κB activation.^[50]^ The studies have demonstrated that acupuncture exerts a downregulatory effect on TNF-α and IL-6 levels, while concurrently upregulating Interleukin-10 levels. Additionally, it facilitates the secretion of BDNF and GDNF, mitigates neuroinflammation, and confers protective effects on cerebral tissues following ischemia/reperfusion injury by inhibiting the toll-like receptor 4 (TLR4)/MyD88/NF-κB pathway.^[83]^

The assembly of NLRP3 inflammasomes initiates the activation of caspase-1, which triggers a cascade of remarkable cellular responses. This subsequently leads to the processing and secretion of IL-1β and IL-18, as well as the cleavage of gasdermin D (GSDMD), thereby facilitating pyroptotic cell death.^[52]^ EA at acupoints *Chize (LU5*), *Hegu (LI4*), *ST36*, and *SP6* can effectively inhibit the expression of NLRP3, precaspase-1, cleaved caspase-1 p20, IL-1β, cleaved IL-1β, and GSDMD protein. The purpose is to attenuate the release of pro-inflammatory mediators following cerebral ischemia/reperfusion injury and suppress neuroinflammation in order to elicit neuroprotective effects.^[84]^ EA at *GV20* and *DU24* can regulate endogenous melatonin secretion, reduce NLRP3, caspase-1, and IL-1β expression, inhibit inflammatory vesicle activation, and decrease pro-inflammatory factor secretion to alleviate cortical inflammation injury and reduce cerebral ischemic injury.^[53]^

These studies have proved that EA mainly improves the inflammatory environment of damaged tissues and reduces neuroinflammation from multiple pathways such as miR-223/NLRP3 pathway, TLR2/NF-κB signaling pathway, TLR4/MyD88/NF-κB pathway, as well as from the gut-brain bi-directional axis, etc. Various mechanisms of inflammation after CIRI can influence and interact with each other. We can consider the interaction between 2 or more mechanisms, and the future direction of exploration is to clarify the interaction between the periphery and the center.

#### 2.2.4. Acupuncture inhibits apoptosis and regulates astrocyte activation

Currently, there are 2 main pathways through which cells undergo apoptosis: the extrinsic pathway and the intrinsic pathway.^[54]^ In response to cerebral ischemia and hypoxia, astrocytes undergo substantial activation, which contributes to the aggravation of brain injury induced by ischemic events.^[85]^ Acupuncture exhibits potential as a therapeutic intervention for various diseases by modulating apoptosis and regulating cellular activation through the inhibition of apoptosis-related factors, while concurrently enhancing the upregulation of anti-apoptotic proteins.

The neurovascular unit (NVU) comprises vascular cells, neuroglia, and neurons. The NVU plays a crucial role in regulating the integrity of cerebral blood flow (CBF), neurovascular repair processes, and the blood-brain barrier, which are essential for maintaining optimal brain functionality. The pathophysiology of ischemic stroke and the process of neurovascular repair are intricately intertwined with the maintenance of homeostasis in the NVU.^[86,87]^ Acupuncture treatment can attenuate the expression of Bad protein, while concurrently enhancing the expression of the anti-apoptotic protein B-cell lymphoma-extra-large. Therefore, this intervention effectively reduces apoptosis in astrocytes, microvascular endothelial cells, and neuronal cells: all crucial components of the neurovascular unit - thereby preserving its integrity after cerebral ischemia.^[88]^

Irisin, a myokine, is secreted from its membrane-bound precursor fibronectin type III domain-containing protein 5 (FNDC5) into the circulation of skeletal muscle. Furthermore, it has the ability to cross the blood-brain barrier and initiate a neuroprotective genetic program within the hippocampus while simultaneously inducing expression of BDNF.^[57]^ After activation of the Peroxisome proliferator-activated receptor gamma coactivators-1-alpha-FNDC5/irisin pathway, it can induce neuroprotective gene programs, including BDNF.^[89]^ The findings of various studies have demonstrated that acupuncture exerts an inhibitory effect on apoptosis and enhances the expression of peroxisome proliferator-activated receptor gamma coactivators-1 alpha, FNDC5, and BDNF proteins within the ischemic cortical regions via activation of the FNDC5/BDNF pathway. Consequently, this mechanism contributes to a reduction in brain I/R damage and plays a pivotal role in neuroprotection.^[90]^

The pivotal role of Cysteine aspartase-3 (Caspase-3) extends to tissue differentiation, regeneration, and neurodevelopment. The role of Caspase-3 as a crucial mediator in neuronal apoptosis has been established, playing an indispensable part in cellular demolition and the formation of apoptotic vesicles.^[58,91]^ EA stimulation at *LI4*, *LU5*, *ST36*, and *SP6* acupoints has been shown to downregulate caspase-3 expression, attenuate apoptosis, and promote nerve regeneration following CIRI.^[92]^

The decrease in arylalkylamine N-acetyltransferase (AANAT) within the ischemic area correlates with neuronal cell death following transient ischemia, while melatonin demonstrates its neuroprotective properties by maintaining AANAT levels specifically in the CA1 region affected by ischemia.^[56]^ EA can reduce neuronal damage and improve neurological and cognitive functions in CIRI by regulating AANAT expression.^[93]^

The PI3K/phosphoprotein kinase B (AKT) signaling pathway has been demonstrated to play a crucial role in protecting neurons against apoptosis or programmed cell death in the context of CIRI.^[55]^ Previous research has demonstrated the efficacy of specific acupoints, including *GV20*, *Yintang (EX-HN3*), and *ST36*, in activating the PI3K/AKT pathway and inducing astrocyte activation to exert neuroprotective effects.^[94]^

In summary, acupuncture exerts an inhibitory effect on apoptosis by modulating the FNDC5/BDNF and PI3K/AKT signaling pathways, facilitating astrocyte activation for neuroprotection, and engaging diverse mechanisms and cellular pathways in the pathogenesis of apoptosis following cerebral ischemia/reperfusion. The precise pathogenesis underlying CIRI remains incompletely elucidated, and the potential benefits of varying levels of cellular activation continue to be a subject for future investigation.

#### 2.2.5. Acupuncture modulates neuroplasticity and promotes nerve regeneration

Mounting evidence suggests that functional recovery following brain injury is primarily driven by the processes of neuroplasticity and nerve regeneration. Therefore, the development of therapeutic protocols that specifically target these pathways is crucial and represents a significant advancement in the treatment of CIRI. Acupuncture can promote axon regeneration, stimulate the proliferation and differentiation of endogenous neural stem cells (NSCs), and facilitate nerve repair by upregulating neuroplasticity-related proteins through the Ras homolog gene family member A/Rho-associated coiled-coil kinase (RhoA/ROCK) signaling pathway and NOTCH1 pathway. The RAGE/RhoA/ROCK pathway is implicated in various neuronal functions, including migration, dendritic development, and axon extension. Inhibition of the RhoA/ROCK signaling cascade can reverse the suppressive effect on axonal growth and promote sprouting as well as functional recovery.^[59]^ EA mitigates the impediment of axonal regeneration in the context of CIRI by diminishing myelin-associated inhibitors and suppressing the RhoA/ROCK signaling pathway. Additionally, it upregulates the expression of growth-associated protein 43 and brain-derived neurotrophic factor following cerebral ischemia-reperfusion, thereby promoting neural regeneration, improving neuronal function, and mitigating cerebral ischemia/reperfusion injury in a rat model.^[95]^

Acupuncture has the potential to enhance axonal regeneration and activation, optimize synaptic structure and function, as well as facilitate neuroplasticity. Astrocytes and microglia likely participate in the modulation of neuroplasticity during acupuncture therapy by producing and releasing various neurotrophic factors, such as BDNF and nerve growth factor.^[14,60]^ Yu et al administered EA to the *EX-HN3* and *GV20* acupoints in order to reverse CIRI-induced alterations in the expression of BDNF, γ-aminobutyric acid type A receptors, Ca^2+^/calmodulin-dependent protein kinase II, N-methyl-D-aspartic acid receptor 1, tyrosine kinase B, α-amino-3-hydroxy-5-methyl-4-isoxazole propionic acid receptor, neuronal nuclei, and postsynaptic density protein 95. This therapeutic intervention modulates neuroprotective mechanisms and enhances synaptic plasticity in pertinent regions of the brain.^[96]^

The Notch signaling pathway epitomizes a canonical mechanism dictating the proliferation and differentiation of neural stem cells. Activation of the Notch1 pathway triggers differentiation of NSCs, thereby effectively promoting neuronal repair and neurogenesis.^[61,97]^ Through activation of the Notch1 pathway, EA can significantly upregulate miR-223 and NESTIN expression, downregulate the expression of PTEN, enhance the proliferation of NSCs, and facilitate functional recovery after CIRI.^[98]^

The aforementioned evidence highlights acupuncture’s potential in regulating neuroplasticity and promoting neural regeneration. However, the natural production and development of NSCs within the central nervous system may not be enough to fully repair damage due to their limited numbers and unfavorable local environment. Therefore, it is imperative to explore adjunctive therapies that can enhance neuronal recovery following CIRI in conjunction with acupuncture.

## 3. The role of acupuncture combined with MSCs in the treatment of CIRI and its mechanism

### 3.1. Effects of MSCs on neural repair after CIRI and its mechanisms

MSCs are a class of pluripotent cell populations possessing stem cell-like characteristics that can be found in various tissues, and they have the ability to differentiate into both neurons and glial cells.^[59]^ MSCs derived from various tissues have gained extensive utilization in the fields of cell therapy, regenerative medicine and tissue engineering owing to their remarkable attributes of pluripotency, immunomodulation and self-renewal.^[62]^ Previous evidence has demonstrated the efficacy of MSCs transplantation in facilitating functional recovery following CIRI, while concurrently reducing infarct size and attenuating apoptotic cell death.^[23]^

Potential mechanisms of mesenchymal stem cell therapy for CIRI include inhibition of neuronal apoptosis, suppression of inflammation, modulation of autophagy, and promotion of angiogenesis. A recent study has demonstrated that Exos lncRNA KLF3 antisense RNA 1 derived from bone marrow MSCs can effectively mitigate cerebral infarction and enhance neurological function. In cells subjected to oxygen glucose deprivation (OGD), it enhances cell survival, suppresses apoptosis, and attenuates inflammation-induced damage as well as the production of reactive oxygen species.

Exosomes derived from bone marrow MSCs were observed to augment the deubiquitinating activity of Silent mating type information regulation 2 homolog 1 (Sirt1), thereby ameliorating inflammatory damage induced by CIRI. This effect was mediated through the KLF3-AS1/miR-206/ubiquitin specific peptidase 22 (USP22) network.^[99]^ Exosome Krüppel-like factor 4 enhances the expression of long noncoding RNA zinc finger antisense (1ncRNA-ZFAS1) by binding to its promoter, thereby leading to the downregulation of N6-methyladenosine levels in dynamin related protein 1, which is implicated in obesity. Consequently, this attenuation of mitochondrial dysfunction contributes to a reduction in neuronal damage observed in cases of ischemic stroke.^[100]^ Recently, the application of small extracellular vesicles secreted by MSCs in cerebral ischemia/reperfusion injury was systematically reviewed, and a meta-analysis showed that small extracellular vesicles derived from MSCs could effectively resolve neurological dysfunction, reduce infarct volume and water content, and attenuate neuronal apoptosis.^[101]^ Administration of stroke serum MSCs after cerebral ischemia/reperfusion can reduce pathological changes, increase neurogenesis, inhibit apoptosis and inflammation, and promote angiogenesis, thus exerting neuroprotective effects.^[102]^ Bone marrow stromal cells-Exos-derived KLF3-AS1 facilitates autophagy through the activation of the ETS variant transcription factor 4/silent information regulator 1 pathway, leading to the alleviation of I/R injury.^[103]^

In conclusion, MSC transplantation therapy for CIRI shows great potential, but large-scale clinical trials are needed to validate it. MSC transplantation therapy mainly exerts neuroprotective effects by inhibiting the inflammatory response and apoptosis, promoting vascular regeneration and neural regeneration. MSCs can migrate to the ischemic area to improve the microenvironment, reduce neuronal apoptosis through immune regulation, and promote nerve tissue repair. Furthermore, the promotion of NSCs proliferation and migration can also be facilitated by MSCs.^[104]^ However, some studies have shown that the low survival rate of MSCs due to the unfavorable microenvironment after injury prevents MSCs located in lesions from traveling to the damaged brain tissue to differentiate into neurons. Hence, it is crucial to improve the viability and specific maturation of MSCs in order to progress their practical use in CIRI treatment.

### 3.2. Efficacy and mechanism of combined acupuncture/MSC in the treatment of CIRI

In recent years, there has been significant progress in the study of the efficacy and mechanism of acupuncture combined with MSCs for treating neurological disorders such as spinal cord injury, stroke, and sciatic nerve injury.^[105–108]^ Meanwhile, previous research has indicated that EA can facilitate the activation of the nervous system to stimulate the release of MSCs into the peripheral blood, and can increase the level of intermediate MSCs in the circulatory system to help tissue repai.^[109]^

After transplanting MSCs into the brain and electro-acupuncture treatment at *GV20* and *Dazhui* point for 1 week after implantation, BMSC migration from the injection site to the cortex on the lesion side was observed, and the combined treatment of BMSC transplantation and electro-acupuncture attenuated the expression of neuron-specific enolase, when compared with electro-acupuncture group or BMSC group alone and demonstrated that local neuronal damage could be effectively repaired after combined treatment.^[108]^ BDNF and neurotrophin-4/5 have been found to support the survival of neurons and promote growth of synapses.^[110]^ Additionally, the expression of tropomysin-related kinaseB (TrkB) receptor has been linked to increased axonal growth, neuronal survival, and plasticity in neurons.^[111–113]^ Implantation of TrkB gene-transfected MSCs (TrkB-MSCs) into the ischemic hemianopic region resulted in the highest quantity of transplanted MSCs observed in the Trk bone marrow stromal cells + EA group after 30 days of middle cerebral artery occlusion. These cells underwent partial differentiation into immature neuroblasts and astrocytes, with a limited presence of fully developed neuron-like cells. After a period of 60 days following middle cerebral artery occlusion, EA was observed to enhance the differentiation of TrkB-MSCs into mature neuron-like cells. In summary, co-transplantation of TrkB-MSCs with EA resulted in enhanced expression of BDNF and neurotrophin-4/5, thereby improving the survival, migration, and neural differentiation capabilities of TrkB-MSCs. Furthermore, this combined approach exhibited a synergistic effect on motor and cognitive functions post-stroke.^[114]^

Based on the above studies, we draw a conclusion that acupuncture combined with MSC transplantation has a potential impact on the localization of CIRI, the curative effect of combined treatment is better than that of acupuncture and moxibustion alone or MSCs implantation (Table 2). Acupuncture improves the local microenvironment of the injury site and plays a neuroprotective role; transplanted MSCs can secrete a plethora of neurotrophic factors, thereby augmenting the neuroplasticity of acupuncture and promoting axonal growth via paracrine mechanisms, thus circumventing the limitations posed by insufficient endogenous neural stem cells at the site of injury and weak promotion ability of acupuncture; acupuncture combined with MSC transplantation treatment can augment the survival and homing rates of MSCs, as well as direct their differentiation into functional neurons.

**Table 2:** The synergistic effects of acupuncture and MSCs transplantation on neural recovery following cerebral ischemia-reperfusion injury and the underlying mechanisms.

Study	Intervention and acupuncture parameters	Control intervention	Effect indicators	Comparison of effects between groups	Mechanism index
[108]	BMSC, EA (GV 20, GV14; 3 Hz, 1 V, 15 min)	Normal group	Repair neuron injury↑	EA + BMSC > EA > BMSC > MCAO	NSE↓
[114]	HUCB-MSC, EA (GV26, GV20, GV14, CV24, CV4, CV6; 30 Hz disperse waves, 100 Hz dense waves, 5 V, 20 min)	PBS group	mNSS↓	HUCB-MSC + EA > HUCB-MSC > PBS	Numbers of VEGF-positive cells↑

CV4 = Guanyuan, CV6 = Qihai, CV24 = Chengjiang, GV20 = Baihui, GV26 = Shuigou, HUCB-MSC = human umbilical cord blood-derived mesenchymal stem cells, mNSS = Modified Neurological Severity Score, MCAO = middle cerebral artery occlusion, MSC = mesenchymal stem cell, NSE = neuron-specific enolase, PBS = phosphate-buffered saline, PBS group = PBS transplantatio, VEGF = vascular endothelial growth factor.

## 4. Conclusions

**Figure 1.**
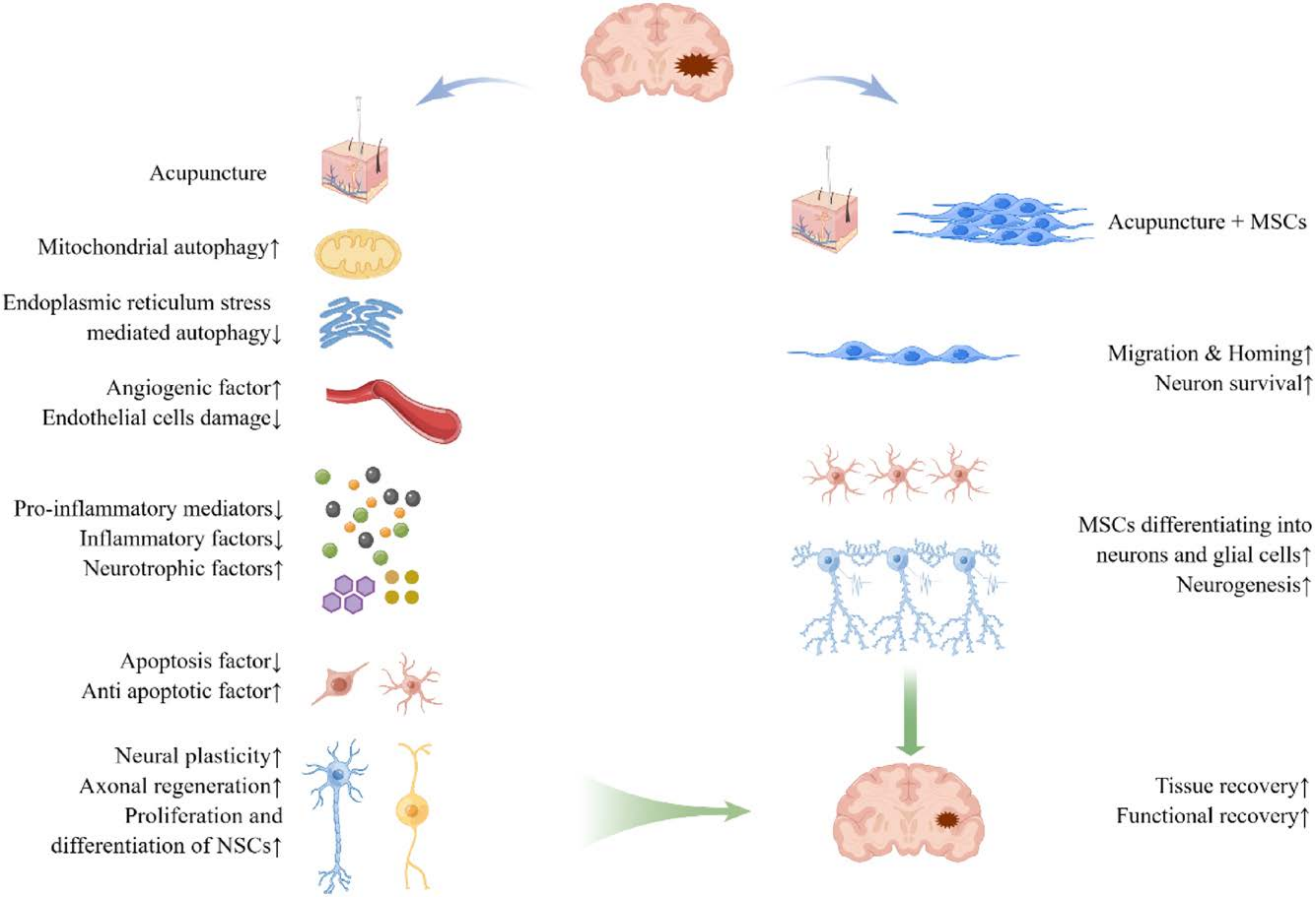
Effects of the combination of acupuncture and MSC transplantation after CIRI and the underlying mechanisms. CIRI = cerebral ischemia-reperfusion injury, MSC = mesenchymal stem cell.

In conclusion, the effects of cerebral ischemia after perfusion injury are severe and not easily recoverable due to the complex pathological changes and limited neuronal regenerative capacity after CIRI. Acupuncture treatment is effective in CIRI (Fig. 1), and the potential mechanisms of acupuncture include regulating autophagy, promoting angiogenesis, inhibiting inflammation, inhibiting apoptosis, regulating cell activation, regulating neuroplasticity, and promoting nerve regeneration, etc. It protects the damaged nerves and promotes the recovery of function through a variety of cellular signaling pathways and related pathway proteins and molecules. MSCs possess the migratory capacity towards the injury site and exhibit neuronal differentiation potential, thereby compensating for the deficiencies in NSCs subsequent to CIRI. Although the transplantation of MSCs can alleviate neuronal apoptosis and promote neurological recovery, their low rates of survival and differentiation, as well as the limited induction of functional neurons, restrict their efficacy for clinical application in CIRI. The combination of acupuncture and MSC transplantation has been shown to yield superior therapeutic outcomes compared to monotherapy, thereby enhancing the survival, homing, and functional differentiation rates of MSCs. Therefore, the combination of acupuncture and MSCs transplantation represents a promising and efficacious approach for future therapeutic interventions in CIRI. It is anticipated that a future multicenter randomized double-blind clinical trial will assess the efficacy of acupuncture in conjunction with MSCs for the treatment of patients suffering from CIRI. Moreover, cutting-edge molecular biology assays, cellular markers, and imaging techniques will be employed to further elucidate the mechanisms underlying neuroprotection, neural regeneration, inflammation inhibition, and other therapeutic effects associated with CIRI.

## Acknowledgments

Thanks to Fig draw for helping with figure production.

## Author contributions

**Data curation:** Huan Li, Jiaxin Zhang, Kewen Ma, Hui Qu, Ruohan Tang.

**Investigation:** Xuesong Ren.

**Methodology:** Chengfei An, Hailun Jiang, Yuzheng Du.

**Resources:** Qi Zhao.

**Writing – original draft:** Huan Li.

**Writing – review & editing:** Jie Ji.
